# Decision fatigue in hospital medicine: A retrospective exploratory evaluation

**DOI:** 10.1002/jhm.70216

**Published:** 2025-10-29

**Authors:** Rachel M. Kruer, LaKeisha Boyd, Lauren Czosnowski, Sarah Jones, Kelsey Perry, Thomas J. Streepey, Areeba Kara

**Affiliations:** ^1^ Inpatient Clinical Pharmacy Services Indiana University Health‐Adult Academic Health Center Indianapolis Indiana USA; ^2^ Department of Biostatistics and Health Data Science Indiana University School of Medicine and Richard M. Fairbanks School of Public Health Indianapolis Indiana USA; ^3^ College of Pharmacy and Health Sciences Butler University Indianapolis Indiana USA; ^4^ Indiana University Health‐Adult Academic Health Center Indianapolis Indiana USA; ^5^ Indiana University School of Medicine Internal Medicine‐ Pediatrics Residency Indianapolis Indiana USA; ^6^ Indiana University Bloomington Bloomington Indiana USA; ^7^ Department of Medicine, Division of General Internal Medicine and Geriatrics Indiana University School of Medicine Indianapolis Indiana USA

## Abstract

**Background:**

“Decision fatigue” (DF) is the deterioration in decision‐making ability stemming from repeated decision making.

**Objective:**

Explore DF over the clinical workblock and across hospitalist and work characteristics.

**Methods:**

Patients seen by hospitalists at two hospitals in January and February 2022 were retrospectively evaluated for events that may reflect DF. Events were selected through multistakeholder discussion (prescription of pharmacologically antagonistic medications, occurrence of hypoglycemia, and potential overuse of blood transfusions, imaging, and penicillin alternatives). For each date, schedules determined the number of days each hospitalist had been on service and charges submitted assessed census and physician familiarity with their team. Charges and ordering data guided event attribution. The primary predictor was the number of days on service, and the outcome was event occurrence. Secondary predictors included hospitalist gender, weekend versus weekday, census, site, and degree of familiarity.

**Results:**

During the study, 43 hospitalists worked 1306 days over 204 working blocks, providing care to 2081 patients over 3122 encounters. Median daily census was 13, median number of consecutive days worked was seven. DF events were not associated with the primary or secondary predictors except census. In the multivariable model, each one unit increase in the number of patients was associated with event occurrence (odds ratio [OR]: 1.107, 95% confidence interval [CI]: 1.022–1.198, *p* = .01). The odds of an event were 1.58 times greater at workloads above the median of 13 patients, compared with workloads ≤13. (*p* = .002).

**Conclusion:**

DF was not detected with an increasing number of days on clinical service. The findings highlight how workloads may threaten care quality.

## Visual Abstract

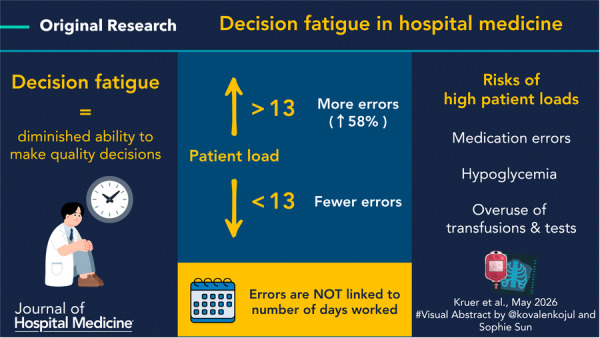

## INTRODUCTION

“Decision fatigue” (DF) is “the impaired ability to make decisions and control behavior as a consequence of repeated acts of decision‐making.”^1^ In healthcare, DF has been implicated in poorer quality of care as the workday progresses—with studies finding higher rates of inappropriate antibiotic prescribing, higher likelihoods of opioid prescribing, and lower rates of breast and colon cancer screenings associated with outpatient appointments later in the day.^2^, ^3^, ^4^

DF is underexplored in hospital medicine as in acute care settingsmost studies have focused on care in the Emergency Department (ED).^5^ In addition to the inherent differences in each setting, hospital medicine poses contexts that may be unique as they relate to the study of DF—including how hospitalists can choose their daily patient rounding order while in both ambulatory and ED workflows, care generally progresses in the order a patient is scheduled or presents. In addition, while DF has largely been described in diurnal patterns, it has been posited that accumulated decisional burden may have harmful effects over the course of a work week.^6^, ^7^, ^8^ As early adopters of the “block” work schedule, where cognitively intense consecutive days are followed by time away from clinical duties, hospitalists may be susceptible to the erosion of decision‐making ability over a clinical block. While the impact of work schedules and discontinuity on mortality, readmissions, and costs have been previously investigated in hospital medicine, DF has not specifically been examined.^9^, ^10^

In this exploratory study, we sought to examine the prevalence of DF over the clinical work block of hospitalists and investigate DF across different characteristics of hospitalists and their work structures.

## METHODS

This manuscript follows the strengthening the reporting of observational studies in epidemiology (STROBE) reporting guidelines for observational studies.^11^ The protocol was reviewed and approved by the Indiana University institutional review board.

### Setting

This study was conducted at two large, urban, Midwestern, academic hospitals belonging to the same health system utilizing data from January and February 2022. The time frame represents a convenience sample following the resumption of post‐coronavirus disease 2019 (COVID‐19) pandemic processes that captures at least two working blocks for each included hospitalist. Hospital 1 is larger than Hospital 2, and the two sites share a single ED. Day‐shift hospitalists care for patients on their assigned teams, admit patients, provide consultations, and assume care for patients downgraded from the intensive care units. They also respond to queries between 7 a.m. and 6 p.m. and are required to be present on site until 3 p.m. at one site and 4 p.m. at the other. On call day‐shift hospitalists are on site until 7 p.m. Arrival times vary between clinicians; however, the expectation is that hospitalists arrive no later than 8:30 a.m. unless assigned to arrive by 7 a.m. Team assignments for physicians include rounding on geographically cohorted units with or without advanced practice providers (APPs), cohorted units with or without learners, and rounding on “at large” teams without APPs or learners. Patient distribution is directed by the unit assignments and accounts for the census of each team. In this setting, there is no plausible reason to expect systematic differences in patient characteristics or workload based on the hospitalist's day on service.

### Participants, data retrieval, predictors, and attribution

All full‐time day‐shift hospitalists who were employed on January 1, 2022, and the patients seen by them were included. Part‐time hospitalists and those who exclusively work as nocturnists were excluded. Workdays where there were fewer than two patients on whom charges were submitted were excluded from the analysis.

A query of the electronic medical record data warehouse retrieved a list of patients on whom included hospitalists had written a note during the study period. For each hospitalization, the age of the patient and length of stay were abstracted in addition to the data required to assess outcomes across the entire hospital stay.

The hospitalist service maintains and archives the work schedule for every clinician. For each included hospitalist, the schedule was retrieved to assign an ordinal number for each day worked (Day 1, Day 2…). Following two or more consecutive days off, the day number was reset to Day 1. The day on service was the primary predictor of interest. When an outcome of interest occurred, it was attributed to the hospitalist who submitted billing on that patient on that date and/or in cases of specific orders—to the physician who placed the order. The number of charges submitted by each physician on each day worked was equated to their census for that day. Examining the charges also allowed us to examine each physician's continuity and familiarity with the patients seen day over day. Per physician per day, we calculated an “unfamiliarity index” by dividing the number of charges submitted on patients who were not billed on the previous day by the total number of charges submitted. An unfamiliarity index of 1 indicates that none of the patients seen by the physician that day were seen by them the previous day, while an index of 0 indicates that all patients were known to that physician from the previous day. Adopting the same rules we employed for numbering workdays, following a break of two or more days, the unfamiliarity index was set to 1 for each Day 1.

### Outcome selection

There is no current consensus on outcomes that best reflect DF in medical settings and previous studies have selected outcomes based on different decision points along the clinical encounter, such as the gathering and interpreting of information and developing diagnostic and therapeutic plans.^5^, ^7^ Pignatiello et al. have previously described how DF may impact decision quality, delay decision making, increase reliance on heuristics, and bias clinicians toward clinically conservative options.^1^ To identify measures that may reflect DF, members of the study team brainstormed to identify clinical scenarios that reflected these consequences. This multi‐stakeholder, consensus‐based approach included perspectives from two clinical pharmacists, an APP, and two physicians. Additional criteria considered included the prevalence of different scenarios, relevance to hospital medicine, ease of electronic extraction, and high‐fidelity assignment of attribution. The final list of selected outcomes is shown in Table 1. If any of these outcomes occurred, the date was marked as having an “event.”

**Table 1:** Description of outcomes selected for study.

Outcome	Definition	Attribution	Link to Decision Fatigue
Use of physiologically contradictory medications	Concomitant prescription of medications that when prescribed together, demonstrate pharmacological antagonism. Pharmacologically antagonist pairs included: antidiarrheals and laxatives, phosphate binders and phosphorus supplementation, potassium binders and potassium supplementation, antihypertensives and agents to raise blood pressure, CNS stimulants and depressants	Physician prescribing the medication that antagonizes the medication already being administered, charts manually reviewed for confirmation.	Poorer quality of decisions
Occurrence of potentially preventable hypoglycemia	First occurrence of “critically low”^a^ blood glucose (< 60 mg/dL) between midnight and 11:59 a.m. or between 12 p.m. and midnight	Before 12 p.m.: attributed to physician billing for patient the day before, 12 p.m. or after attributed to physician billing for patient that day.	Delays in decision making, therapeutic inertia
Potentially guideline nonconcordant blood transfusions	Packed red blood cell transfusion when hemoglobin ≥7 gm/dL	Physician billing on transfusion date	Choosing default conservative option
Potential over avoidance of penicillin	Prescription of aztreonam, meropenem, or imipenem in those with penicillin allergy	Physician prescribing aztreonam, meropenem, or imipenem	Choosing default conservative option
Potential over use of head CT	Noncontrasted head CT with order indication of: altered mental status, encephalopathy, confusion, delirium, or syncope	Physician ordering scan	Choosing default conservative option

Abbreviations: CNS, central nervous system; CT, computed tomography.

^a^ Threshold at which notification to the clinical team is required at our institution.

### Analysis

The primary predictor of interest was the day on service, and the primary outcome of interest was the occurrence of an event. We explored secondary predictors including hospitalist gender, whether the workday was a weekend or weekday, the unfamiliarity index, census, and hospital site. These variables were explored as previous research indicates that physician gender, differences in resource availability on weekends versus weekdays, continuity of inpatient care, and workload may also impact outcomes.^9^, ^10^, ^12^, ^13^, ^14^ Hospital site was explored to confirm that there were no unmeasured site‐specific differences.

Descriptive statistics were generated to characterize the study population and examine workday characteristics. Frequency and percentage values were given for categorical measures while continuous variables were measured using mean, median, standard deviation, inter‐quartile range, and range values.

Logistic regression models were fit to assess the relationship between primary and secondary predictors and the occurrence of DF events within a workday. The outcome for the bivariate and multivariable models was a compound variable that was “yes” if there was an event present and “no” if no event occurred. Each model included random effects that nested hospitalist within their working block to account for hospitalists working numerous shifts over several working blocks. A working block was defined as a block of days worked together with a minimum of two days off between workdays.

Once census was found to be associated with DF events, to be able to assess the application of this finding to the clinical environment, additional sensitivity analyses were conducted using the median patient workload of 13 as a cut point. The multivariable logistic regression analysis was repeated replacing the continuous patient workload variable with a binary variable for workloads > 13 and ≤ 13.

A 5% significance level was used for all analyses, and analyses were performed using SAS version 9.4 (SAS Institute, Inc.) and RStudio version 4.2.3 (RStudio).

## RESULTS

Between January 1 and February 28, 2022, 43 hospitalists worked 1306 days over 204 blocks and provided care to 2081 patients during 3122 encounters. Fifteen (34.9%) hospitalists were female, and the median number of consecutive days worked was 7 with a median of 8 days off between working blocks. The median census was 13 patients per day with a median unfamiliarity index of 0.18. Patients' mean age was 57.5 years. Table 2 summarizes hospitalist, patient and hospitalization, workday, and work block characteristics.

**Table 2:** Hospitalist, patient, hospitalization, workday, and work block characteristics.

Hospitalists	
Total	43
Sex, female, *n* (%)	15 (34.9)
Number of hospitalists for whom ≥50% workdays had workload >13 patients, *n* (%)	6 (14.0)
Number of hospitalists who had at least one work block where ≥50% of workdays had a workload > 13 patients, *n* (%)	15 (34.9)

**Workdays**	
Total	1306
Site, *n* (%)	
Hospital 1	948 (72.6)
Hospital 2	358 (27.4)
Census per day (number of patients billed on), mean, median (SD)	12.6, 13 (1.9)
New patients per day, median (IQR)	2.0 (1.0–4.0)
Unfamiliarity index per day, median (IQR)	0.18 (0.1–0.3)
Weekend, *n* (%)	403 (30.9)

**Work block**	
Total	204
Number of consecutive days worked, median (IQR)	7.0 (6.0–8.0)
Number of consecutive days off, median (IQR)	8.0 (6.0–8.0)

**Patients**	
Total	2081
Age, years, mean (SD)	57.5 (17.7)

**Encounters**	
Total	3122
Inpatient encounter type, *n* (%)	2858 (91.5)
Length of stay, days, median (IQR)	7.0 (4–12)
Discharge disposition^a^ (*n*%)	
Home or self‐care	1787 (57)
Skilled nursing facility	519 (16)
Home health care organization	249 (8)
Rehabilitation facility	183 (5.9)
Hospice	133 (4.3)
Other	246 (7.8)
Inpatient mortality, *n* (%)	91 (2.9)

Abbreviations: IQR, interquartile range; SD, standard deviation.

^a^ Data missing from five encounters.

Of 1306 examined workdays across 43 hospitalists, no events occurred for the majority (*n* = 1008, 77.2%) of days. Hypoglycemia was the most frequently noted event (Supporting Information S1: Table 1).

In unadjusted comparisons, there were no differences by the hospital site, unfamiliarity index, whether it was a weekend or weekday, and event occurrence (Supporting Information S1: Table 2).

In both bivariate and multivariable analyses, the occurrence of an event was not statistically significantly associated with the number of days worked, hospitalist gender, weekend versus weekday, unfamiliarity index or hospital site. However, in both models, an increase in census was associated with a higher number of events. In the bivariate model, for each 1‐patient increase in the number of patients, the odds of a DF event increased by 10.7% (*p = *.011). Similarly, in the multivariable model, each one‐unit increase in the number of patients was significantly associated with the occurrence of an event (odds ratio [OR]: 1.107, 95% confidence interval [CI]: 1.022–1.198, *p* = .012) (Table 3).

**Table 3:** Logistic regression analyses for the presence of event.

	Bivariate results		Multivariable results	
Predictor	OR (95% CI)	*p* value	OR (95% CI)	*p* value
Day number: Each 1‐day increase	1.009 (0.954, 1.067)	.750	1.020 (0.955, 1.090)	.555
Hospitalist gender: Female versus male	1.155 (0.853, 1.564)	.353	1.148 (0.855, 1.542)	.359
Site: Hospital 1 versus Hospital 2	0.820 (0.599, 1.121)	.213	0.779 (0.570, 1.066)	.119
Weekend: Yes versus No	0.941 (0.704, 1.259)	.683	1.036 (0.766, 1.402)	.818
Unfamiliarity Index: Each one‐unit increase	1.073 (0.729, 1.578)	.722	1.157 (0.747, 1.793)	.513
Census: Each one‐patient increase	1.107 (1.024, 1.196)	.011	1.107 (1.022, 1.198)	.012

Abbreviations: CI, confidence interval; OR, odds ratio.

A multivariable sensitivity analysis revealed that the odds of a DF event were 1.583 times greater when the census was more than 13 patients when compared with census ≤13 (*p* = .002, CI: 1.180, 2.124) (Supporting Information S1: Table 3).

## DISCUSSION

In this exploratory, retrospective evaluation, we found no association between the number of days worked and DF events among hospitalists. Of the variables examined, only the daily patient census was associated with a higher odds of having a DF event.

DF is understudied among hospitalists and has largely been assessed in diurnal patterns.^5^, ^7^ This study therefore represents an early exploration of DF in hospital medicine and for time frames relevant to the common hospitalist schedule of working a clinical “block” followed by time off. We did not detect an increase in DF events over the course of a hospitalist's working block. Our sample size was limited by the number of hospitalists at our institution, and we may have been underpowered to detect small differences.

It is instructive to consider our results in the context of previous literature. It is possible that we did not detect changes in DF events with the progression of the workblock as the time between the end and start of the workday is enough to replenish decision‐making reserve; however, even diurnal DF is inconsistently detected in inpatient settings.^5^, ^7^ It has been postulated that over the course of a shift, there may be a “tuning up” and improvement in clinical decision making with each patient seen that counteracts any decline related to DF.^15^ It is possible that a similar phenomenon occurs among hospitalists and improved judgement, heightened attention, and self‐awareness of fatigue over the course of a clinical block may mitigate the impact of the fatigue itself.

Situational awareness can be summarized as the “(1) the perception of data elements, (2) the comprehension of their meaning in context, and (3) the projection of their status in the near future.”^16^ Hospitalists may develop situational awareness related to fatigue and deploy counter measures accordingly. Situational awareness may also explain the lack of association between the unfamiliarity index and event occurrence. Anecdotally, many hospitalists dread the first day on service when they must learn a large amount of information about the patients on their teams. Yet, familiarity did not appear to impact the probability of a DF event. In this retrospective evaluation, we did not measure self‐reported ratings of DF, the perceived effort or time taken to complete care, or any cognitive counter measures deployed by hospitalists—and can only generate hypotheses which deserve further study. As our understanding of these concepts evolves, it will allow us to craft optimized, evidence‐based schedules that balance the tension between the risks of discontinuity and any potential contribution from DF.

Several studies addressing hospitalist workloads suggest that increasing workloads may be associated with increases in patient length of stays, with higher effect sizes reported in earlier hospital medicine literature and mediated by patient acuity, hospital occupancy, and hospitalist experience.^14^, ^17^, ^18^, ^19^, ^20^ Increasing workloads may also be associated with threats to patient safety.^21^, ^22^ These studies are often conducted in settings where workloads are lower than the national average and many predate the COVID‐19 pandemic following which patient acuity among hospitalized patients has been rising.^23^ According to the 2023 State of Hospital Medicine survey, more than a third (35%) of responding sites reported a daily census of 16 or more patients and only 17.9% of those handling this workload strongly agreed with the statement: “my average patient load is safe.”^24^ Our findings align with these self‐reported risks perceived by individual hospitalists. In our setting, with a median daily workload of 13 patients, the odds of there being an event was 58% higher when there were more than 13 patients seen in the day versus 13 or fewer. As hospital medicine groups develop, sustain, and advocate for their programs they should consider the implications of our findings and those of other analyses that suggest that lowering the workload may result in substantial cost savings through length of stay reductions.^25^ As hospital medicine expands, definitions of success should evolve to reflect metrics beyond productivity, and workload targets should consider the nuanced balances between the proposed workload, the context of the work environment and hospitalist workforce characteristics, and the desired outcomes in our missions of patient care, education, wellness, and institutional success.^26^

Our work has limitations. In addition to the power considerations discussed, as a single‐center study, our findings may not be generalizable. Our data spans 2 months, and temporal and seasonal trends may be missed. As the work is retrospective, the context of the decisions is not known. Billing data may not reflect all patients hospitalists may have expended cognitive capacity on without associated billing (e.g., providing support to APPs or discussing patients with the transfer center). There are no prespecified outcomes that are known to best measure DF, and while we selected a range of decisions that reflect daily clinical practice, studying different outcomes such as decisions related to low‐value practices in hospital medicine may have yielded different results.^27^ Future work should seek to test and validate different scenarios to better define and measure DF in the inpatient setting. As an exploratory study, we focused on DF detection—and did not delve into all possible antecedents, attributes, and consequences that may be important in advancing DF knowledge.^5^

We did not detect an increase in the likelihood of a DF event over the course of a clinical work block among hospitalists. We did find an association between higher patient loads and the occurrence of DF events. The downstream consequences of these events may coalesce to impact more complex outcomes. Hospital medicine groups should evaluate their patient workload targets against their goals for patient safety, efficiency, clinician satisfaction, and institutional success—as less may be more.

## CONFLICT OF INTERESTS STATEMENT

The authors declare no conflicts of interest.

## Supporting information

Revision 3 Supplemental Tables Changes accepted.
