# Supplementary material for: Decision fatigue in hospital medicine: A retrospective exploratory evaluation
Source: J Hosp Med. 2025 Oct 29;21(5):506–11. doi: 10.1002/jhm.70216 (PMC13136919; doi:10.1002/jhm.70216)
Supplement: Supplementary file 1 — Revision 3 Supplemental Tables Changes accepted. [file JHM-21-506-s001.docx]

**Supplemental Table 1: Distribution of event types and occurrence over workdays**

| Total number of workdays | 1306 |
| --- | --- |
| Total number of events | 372 |
| Number of events per day | Incidence over total examined days (number of workdays with that number of occurrence(s)/total number of workdays) |
| 0 events | 1008 (77.2%) |
| 1 event | 237 (18.2%) |
| 2 events | 49 (3.8%) |
| 3 events | 11 (0.8%) |
| 4 events | 1 (0.1%) |
| Hypoglycemia |  |
| Total number of hypoglycemia events | 220 |
|  | Incidence over total examined days (number of workdays with that number of occurrence(s)/total number of workdays) |
| 1 event | 155 (11.9%) |
| 2 events | 25 (1.9%) |
| 3 events | 5 (0.4%) |
| Physiologically contradictory medications |  |
| Total number of physiologically contradictory medication events | 94 |
|  | Incidence over total examined days (number of workdays with that number of occurrence(s)/total number of workdays) |
| Anti diarrheal contradictions (n=46) |  |
| 1 event | 42 (3.2%) |
| 2 events | 2 (0.2%) |
| CNS stimulant contradictions (n=5) |  |
| 1 event | 5 (0.4%) |
| Cation exchange resin contradictions (n=1) |  |
| 1 event | 1 (0.1%) |
| Phosphorus binder contradictions (n=1) |  |
| 1 event | 1 (0.1%) |
| Anti-hypertensive contradictions (n=41) |  |
| 1 event | 41 (3.1%) |
| Potential over avoidance of Penicillin |  |
| Total number of potential over avoidance of penicillin events | 15 |
|  | Incidence over total examined days (number of workdays with that number of occurrence(s)/total number of workdays) |
| 1 event | 15 (1.2%) |
| Potential over use of Head CTs |  |
| Total number of potential over use of Head CT events | 26 |
|  | Incidence over total examined days (number of workdays with that number of occurrence(s)/total number of workdays) |
| 1 event | 26 (2.0%) |
| Potentially guideline non-concordant blood transfusion |  |
| Total number of potentially guideline non-concordant blood transfusions | 17 |
|  | Incidence over total examined days (number of workdays with that number of occurrence(s)/total number of workdays) |
| 1 event | 17 (1.3%) |

Abbreviations: CNS= central nervous system, CT= computed tomography

**Supplemental Table 2 Shift characteristics by the presence of an event.**

|  | **No Events**  **(N=1008)** | **One or More Events**  **(N=298)** | **Total**  **(N=1306)** | **p-value** |
| --- | --- | --- | --- | --- |
| **Site, n (%)** |  |  |  | 0.169 |
| Hospital 1 | 741 (73.51%) | 207 (69.46%) | 948 (72.59%) |  |
| Hospital 2 | 267 (26.49%) | 91 (30.54%) | 358 (27.41%) |  |
| **Number of patients on whom charges submitted** |  |  |  | 0.003 |
| N-Missing | 9 | 0 | 9 |  |
| Mean (SD) | 12.48 (1.92) | 12.85 (1.95) | 12.56 (1.93) |  |
| Median (IQR) | 13.00 (11.00, 14.00) | 13.00 (12.00, 14.00) | 13.00 (11.00, 14.00) |  |
| Range | 5.00 - 19.00 | 7.00 - 19.00 | 5.00 - 19.00 |  |
| **Weekend shift, n (%)** |  |  |  | 0.673 |
| Yes | 314 (31.15%) | 89 (29.87%) | 403 (30.86%) |  |
| **Unfamiliarity index** |  |  |  | 0.546 |
| N-Missing | 9 | 0 | 9 |  |
| Mean (SD) | 0.30 (0.33) | 0.31 (0.33) | 0.31 (0.33) |  |
| Median (IQR) | 0.18 (0.09, 0.31) | 0.20 (0.09, 0.31) | 0.18 (0.09, 0.31) |  |
| Range | 0.00 - 1.00 | 0.00 - 1.00 | 0.00 - 1.00 |  |

**Supplementary Table 3. Sensitivity analysis with cut-point for number of patients in multivariable analysis.**

| **Predictor** | **Estimate (95% CI)** | **p-value** |
| --- | --- | --- |
| Day Number: Each 1-Day Increase | 1.019 (0.953, 1.090) | 0.577 |
| Gender: Female vs. Male | 1.158 (0.861, 1.558) | 0.333 |
| Site: Hospital 1 vs Hospital 2 | 0.770 (0.563, 1.054) | 0.103 |
| Weekend: Yes vs. No | 1.032 (0.764, 1.395) | 0.836 |
| Unfamiliarity Index: Each 1-Unit Increase | 1.172 (0.758, 1.814) | 0.475 |
| Number of Pats: >13 vs. <=13 | 1.583 (1.180, 2.124) | 0.002* |
